# Inhibition of TGF-β in Tubule Cells Increases Respiration via Upregulation of Respiratory Genes

**DOI:** 10.1177/19373341251389007

**Published:** 2025-10-22

**Authors:** Kuniko Hunter, Shuvo Roy, Alissa Ice, Rachel C. Evans, Roy Zent, William H. Fissell

**Affiliations:** 1 Department of Biomedical Engineering, Vanderbilt University, Nashville, Tennessee, USA.; 2 Division of Nephrology and Hypertension, Vanderbilt University Medical Center, Nashville, Tennessee, USA.; 3 Bioengineering and Therapeutic Sciences, University of California, San Francisco, California, USA.

**Keywords:** cell culture stress, mitochondria, oxidative phosphorylation, renal tubule cell, TGF-β

## Abstract

**Background:**

Renal tubule cells lose differentiated characteristics in artificial culture, limiting their application in medical research and cell therapy. We previously showed that adding inhibitors of transforming growth factor-β (TGF-β) signaling to cell culture media increased specific transport functions characteristic of differentiated tubule cells. Transport in proximal tubule cells is energetically demanding; *in vivo*, these cells rely primarily on oxidative phosphorylation of fatty acids for adenosine triphosphate (ATP) generation. We examined whether TGF-β inhibition, with or without metformin, altered glycolysis and oxidative phosphorylation compared with standard culture conditions.

**Approach:**

Primary renal tubule cells (PRTC) were cultured with or without an inhibitor of TGF-β receptor I and with or without metformin in a 2 × 2 factorial design. First, expression of proteins in fatty acid transport and the electron transport chain was compared between conditions. The relative contributions of glycolysis and oxidative phosphorylation to ATP generation were assessed by extracellular acidification rate (ECAR) and oxygen consumption rate (OCR). We also tested substrate-specific contributions using inhibitors of pyruvate, glutamine, and carnitine mitochondrial entry. Finally, OCR and transport were measured after 48 weeks in culture to determine durability of culture phenotype.

**Results:**

Metformin and SB431542 increased expression and phosphorylation of proteins in the electron transport chain and involved in fatty acid transport. Metformin and TGF-β inhibition increased oxidative phosphorylation. Metformin decreased glucose dependency, while combination with TGF-β inhibition increased fatty acid dependency. Differences in OCR and transport between treatment conditions persisted at 48 weeks in culture.

**Discussion:**

Renal tubule cell transport is energetically demanding, so cellular differentiation requires matching increases in energetic machinery. We found that metformin and inhibition of TGF-β increased oxygen consumption and utilization of fatty acids in cultured primary tubule cells. These data support the hypothesis that TGF-β inhibition *in vitro* not only increases expression of a broad array of transporters characteristic of the proximal tubule, as we previously showed, but also improves the supply of energy to support active transport.

**Impact Statement:**

A major challenge in tissue engineering is quantitative fidelity of the engineered entity to its *in vivo* counterpart, which is particularly challenging in hepatocytes, islet cells, and kidney cells. Here, we show that blocking transforming growth factor β not only improves renal tubule cell function as we previously found but also upregulates the machinery of oxidative phosphorylation to provide the ATP-borne energy to support apicobasal transport.

## Introduction

Kidney failure imposes a substantial burden on patients and caregivers and consumes disproportionately high health care resources relative to its prevalence in industrialized countries. The best treatment, organ transplantation, is severely limited by scarcity of donor organs, while dialysis remains an incomplete and resource-intensive therapy. For these reasons, tissue engineering of kidney replacement therapy has the potential to mitigate the personal and societal harm of kidney failure. Kidneys continuously excrete a large mass of wastes to ensure that concentrations remain sufficiently low enough to prevent intoxication. To process hundreds of liters of blood per day while maintaining extracellular fluid balance, renal tubule epithelial cells actively transport salt and water, concentrating wastes into a small fluid volume.^1^ This is highly energy-intensive, making the kidneys the body’s second most energy-demanding organ in terms of both mitochondrial content and oxygen consumption. About 95% of ATP in a healthy proximal tubule epithelial cell is produced by oxidative phosphorylation, the oxygen-consuming process that couples ATP synthesis to the movement of electrons through the mitochondrial electron transport chain (ETC).^2^ In contrast, when renal proximal tubule epithelial cells are cultured *in vitro*, they tend to metabolize glucose using anaerobic glycolysis. This switch from oxidative phosphorylation to glycolysis is a component of a broader phenotypic attenuation inherent in *in vitro* culture, collectively known as cell culture stress. We previously found that inhibition of transforming growth factor-β (TGF-β) in cultured renal tubule cells promotes apicobasal fluid transport, glucose uptake, organic anion excretion, and apical acid extrusion.^3–5^ TGF-β is a pleiotropic cytokine that governs development, fibrosis, and oncogenesis across many tissue types.^6–8^ TGF-β reduces the transcription of Tricarboxylic Acid Cycle (TCA)-related enzymes in numerous cell types and induces mitochondrial fractionation in renal tubule epithelial cells.^9–13^

The addition of an AMP-activated protein kinase (AMPK) activator, metformin, increases the effects of TGF-β inhibition on tubule cell function.^3,4^ The effects of TGF-β inhibition and metformin on *in vitro* differentiation appeared to span an array of gene expression and transport functions, motivating inquiry into the effects of these small molecules on cellular energy generation. We hypothesized that metformin and TGF-β inhibition enhance oxidative phosphorylation through activity of the transcription factor Peroxisome proliferator-activated receptor-γ coactivator-1α (PGC-1α). PGC-1α enhances oxidative phosphorylation by increasing pyruvate dehydrogenase (PDH) expression and mitochondrial DNA synthesis and altering voltage-dependent anion channel (VDAC) activity.^14,15^

## Materials and Methods

### Cell culture

Primary human renal tubule epithelial cells (PRTC) were obtained from Innovative Biotherapies (Ann Arbor, MI) and Lonza (Cat CC-2553, Basel, Switzerland). Cells were maintained in a 1:1 ratio of glucose-free Dulbecco's Modified Eagle's Medium (DMEM)/F12 media with supplements as previously described.^4^ Cells were seeded onto polycarbonate Transwell cell culture inserts (Corning Inc., Corning, NY) or polystyrene Seahorse 96-well tissue culture plates (Agilent Technologies, Santa Clara, CA) at a density of 100,000 cells cm^−2^ and incubated overnight. After 24 h, cell culture plates were moved to an orbital shaker to simulate physiological shear stress (2 dyne cm^−2^). After 1 week in culture for 96-well plates and 2 weeks in culture for tissue culture supports, cells were supplemented apically with AMPK activator metformin (200 µM; Cayman Chemical, Ann Arbor, MI), TGF-β receptor I inhibitor SB431542 (10 µM; Cayman Chemical), or a combination of both. Control cells were treated with dimethyl sulfoxide vehicle control (1 µL/mL). Cells were cultured with treatments for 4 weeks prior to staining and mRNA and protein harvest.

### Apicobasal transport

PRTC were cultured on Transwell inserts as described above. After 4 weeks of treatment (cells from Innovative Biotherapies) or a total of 46 weeks (cells from Lonza), cultures were aspirated and changed to fresh media. Twenty-hour hours later, apical media was aspirated and weighed. Three blank wells with the porous membrane occluded with epoxy were used as evaporative controls. Volume transport was assessed by determining the differences between initial volumes and final volumes, as estimated by weight, and correcting for evaporation.

### Quantitative RT-PCR

RNA was extracted using an Rneasy kit with on-column Dnase I digestion (Qiagen, Valencia, CA). First-strand cDNA was synthesized using a high-capacity cDNA reverse transcription kit (Applied Biosystems, Carlsbad, CA) according to the manufacturer’s instructions. Primers (Supplemental Data) were selected from the Harvard-Massachusetts General Hospital Primerbank or generated using PrimerBLAST (NCBI, Bethesda, MD) and purchased from Sigma-Aldrich (St. Louis, MO). Reactions were performed using a CFX96 Real-Time PCR System (Bio-Rad, Hercules, CA) under standard running conditions with Sso Advanced Supermix (Bio-Rad). mRNA transcript counts were normalized to the housekeeping gene glyceraldehyde-3-phosphate dehydrogenase or β-actin.

### Immunoblotting

Cells were rinsed once with phosphate-buffered saline (PBS) and lysed in ice-cold lysis buffer (PBS containing 0.05% sodium dodecyl sulfate [SDS], protease inhibitor, and phosphatase inhibitor). Cells were then scraped, collected, and sheared. Total protein concentration was determined using a bicinchoninic acid assay (Pierce Biotechnology, Waltham, MA). Cell lysates were resolved on Mini-PROTEAN TGX 4–20% polyacrylamide gels (Bio-Rad), at a concentration of 10 µg protein per lane, under reducing conditions with SDS-glycine running buffer. Proteins were transferred to a nitrocellulose membrane using a wet blotter. Blots were fully dried and blocked with 5% (wt/vol) bovine serum albumin (BSA) in Tris-buffered saline with 0.1% Tween-20 (TBST). Membranes were incubated overnight at 4°C with primary antibody (Supplemental Data) diluted in 5% BSA in TBST and detected using the appropriate near-infrared secondary antibody. Membranes were imaged using near-infrared fluorescence on an Odyssey CLx (LI-COR, Lincoln, NE). Band intensities were measured and normalized to β-actin using ImageStudio (LI-COR) to control for variation in loading. For each protein target, individual band density was normalized to the mean density of all corresponding bands in the relevant control lanes.

### Seahorse respirometry

Cell oxygen consumption (OCR) and extracellular acidification rates (ECAR) were measured using a Seahorse Xfe96 (Agilent, Santa Clara, CA) after 4 weeks of treatments (cells from Innovative Biotherapies) or using a Seahorse Xfe24 after 46 weeks (cells from Lonza). For the mitochondrial stress test and substrate dependency tests, OCR and ECAR were assessed after 4 weeks of treatments. Baseline OCR and ECAR levels were obtained from an average of three independent measurements per well. Following completion of the assays, cells were washed with PBS and fixed with 4% paraformaldehyde on ice for 20 min. Substrate dependency was determined by quantifying the change in OCR following addition of the target substrate inhibitor, normalized to the total change in OCR after sequential addition of all inhibitors. Cells were mounted with VectaShield containing diamidino-2-phenylindole dihydrochloride (ThermoFisher, Waltham, MA), and nuclei were counted using an ImageXpress automated imaging system to obtain cell count (Molecular Devices, San Jose, CA).

Cells grown in 96-well Seahorse plates were glucose-starved for 1 h prior to the glycolytic stress test. For 46-week respiration experiments, cells were grown on permeable supports and enzymatically detached into single-cell suspensions, then placed in 24-well Seahorse islet isolation plates as follows. Each well of the XF24 cell culture microplate was coated with Corning Celltak (39.3 µL of Celltak added to 1960.7 µL of purified water). Fifty microliters were added to each well and incubated for 20 min at room temperature. Each well was then washed twice with deionized water. The transwell plate containing Lonza renal proximal tubule epithelal cell was removed from the incubator after 46 weeks in culture, cell culture media was aspirated, and each well was washed with PBS. Two hundred microliters of protease from *Bacillus licheniformis* 10 mg/mL stock were added to each well for about 5 min. Cell and protease mixture was aspirated and placed in individual microcentrifuge tubes and spun at 16,000 RCM for 2 min. The supernatant was carefully removed, and cells were resuspended in 100 µL Seahorse DMEM media containing 1 mM sodium pyruvate, 2 mM l-glutamine, and 10 mM glucose. 10 µL of cell mixture was removed and mixed 1:1 with Trypan Blue. Ten microliters of cell and trypan blue were removed and placed in a hemocytometer for counting. Each of the four quadrants was counted, and an average was taken. The average was then multiplied by 2 × 10^4 × 0.09 for a final cell count for each well. The remaining 90 µL of resuspended cells were added to the Seahorse cell culture microplate and centrifuged at 200 G for 1 min and then incubated in a non-carbon dioxide (CO_2_) incubator at 37°C for 30 min. Each well was then brought up to 400 µL of Seahorse DMEM media containing 1 mM sodium pyruvate, 2 mM l-glutamine, and 10 mM glucose. Each plate was then returned to the non-CO_2_ incubator at 37°C for an additional 15–25 min until the Seahorse instrument was ready.

Media used in all respirometry experiments lacked metformin or SB431542. For mitochondrial stress tests, oligomycin (2 µM), carbonyl cyanide m-chlorophenylhydrazone (CCCP, 12 µM), rotenone (0.5 µM), and antimycin A (0.5 µM) were injected to determine cellular mitochondrial function. For these experiments, three serial OCR measurements were taken to determine OCR in the presence of each respiratory inhibitor. For substrate dependency experiments, glutamine oxidation inhibitor bis-2-(5-phenylacetamido-1,2,4-thiadiazol-2-yl)ethyl sulfide (10 µM), fatty acid oxidation inhibitor etomoxir (100 µM), and glucose oxidation inhibitor UK5099 (10 µM) were injected to determine the cellular response to substrate inhibition. For these experiments, five serial OCR/ECAR measurements were taken to determine OCR/ECAR in the presence of each respiratory inhibitor. Substrate dependency was calculated by determining the change in OCR caused by the target substrate inhibitor and dividing it by the total change in OCR after administration of all substrate inhibitors. OCR and ECAR values were then normalized to cell count for all experiments.

### Immunofluorescence imaging

Cells were washed with ice-cold PBS and fixed with 4% paraformaldehyde for 20 min on ice, then permeabilized with 0.1% Triton-X in PBS for 10 min, and then washed three times with PBS. Cells were blocked for 1 h at room temperature with 5% goat serum and 1% BSA in PBS, then incubated with primary antibody (1:200) at room temperature for 1 h. Cells were then washed three times with PBS, then incubated for 1 h in the dark with fluor-conjugated secondary antibody (1:10,000) for 1 h at room temperature. Antibody sources are listed in the Supplemental Data. Cells were then washed overnight in PBS and then fixed with mounting medium. Images were obtained with a Zeiss LSM 710 confocal microscope using Zen Black (Zeiss, Oberkochen, Germany).

### Quantification and statistical analysis

All results are expressed as mean ± standard error of the mean of a minimum of three independent experiments. Differences in means were assessed with one-way analysis of variance tests using Dunnett’s T3 multiple comparisons test with a single pooled variance computed for each comparison using Prism 9 (GraphPad, San Diego, CA) or RStudio v2024.12.0 + 467 (Posit Software, Boston, MA). Statistical significance was accepted at a level of *p* < 0.05 with Bonferroni corrections for multiple comparisons where appropriate.

## Experiment

### Metformin and SB431542 increase apicobasal volume transport

We previously showed that when cultured according to our protocols, primary renal proximal tubule epithelial cells (Lonza Catalog CC-2553) have diuretic inhibitable volume transport, phlorizin-inhibitable glucose transport, probenecid-inhibitable organic acid secretion, and other functions characteristic of proximal tubule cells.^4^ In addition, indirect immunofluorescence imaging of these cells shows NHE3, Megalin, and SGLT2 (Supplementary Fig. S1). After 4 weeks in culture, apicobasal fluid transport by control and treated PRTC was measured. Apicobasal fluid transport was greater in cells treated with metformin, SB431542, or both (control: 70.67 ± 3.749 µL cm^−2^ day^−1^ vs metformin: 74.34 ± 3.876 µL cm^−2^ day^−1^ [*p* = 0.0041]; SB431542: 95.53 ± 3.666 µL cm^−2^ day^−1^ [*p* < 0.0001]; combination: 137.1 ± 1.118 [*p* < 0.0001]; Figure 1B, open circles). Similar effect was measured in PRTC at week 46 (113.1 ± 4.4 µL cm^−2^ day^−1^ versus 44.3 ± 2.9 µL cm^−2^ day^−1^, *p* < 0.0005), Figure 1B, filled circles). Basolateral dextran was lower in combination-treated cells than in control cells (ratio basal:apical 0.12 ± 0.007 versus 0.43 ± 0.02, *p* < 0.0008), consistent with improved barrier function in treated versus control cells.

**FIG. 1.**
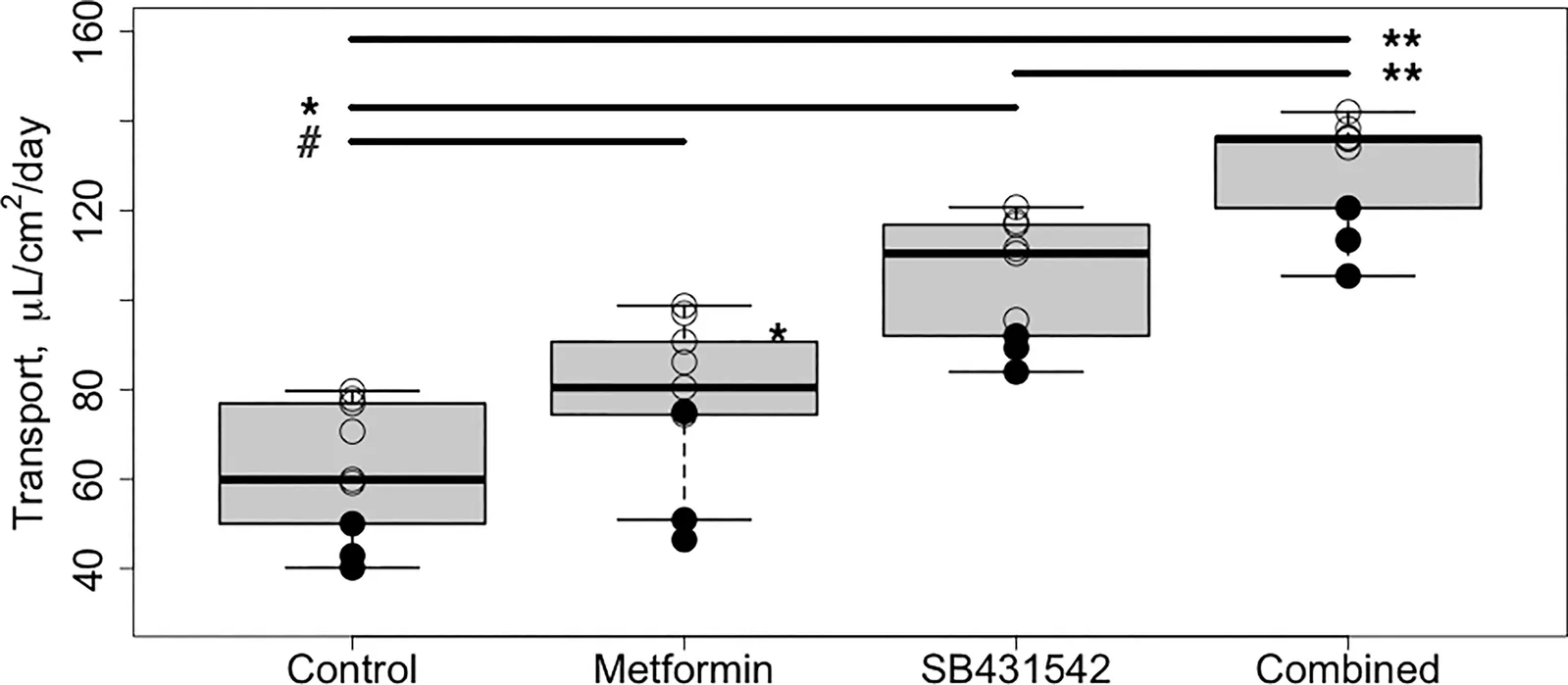
Metformin and SB431542 increase apicobasal transport in renal tubule epithelial cells. Apicobasal fluid transport in the presence of metformin, SB431542, and combination treatment (*n* = 6 at 2.5 weeks, *open circles*; *n* = 3 at 46 weeks, *filled circles*). ^#^*p* < 0.01 for 2.5 weeks only, *p < 0.001, **p < 0.0001 for all cells pooled results, and *p* < 0.001 for 2.5 weeks or 46 weeks individually.

### Metformin and SB431542 increase transcription of genes in the ETC

Mitochondrial biogenesis requires the coordinated expression of nuclear- and mitochondrial-encoded subunits of the mitochondrial respiratory complexes, so we examined whether treatments altered the expression of mitochondrial enzymes.^16^ Metformin and combination treatments increased transcription of mitochondrially encoded complex I (mt-ND1) and decreased transcription of nuclear-encoded uncoupling protein 2 (UCP2), but not SB431542 alone (Fig. 2A, B). SB431542 increased expression of nuclear-encoded complex III (UQCRC) (Fig. 2C). SB431542 and combination treatment increased nuclear-encoded complex I (NduFB8) (Fig. 2D). Mitochondrially encoded complex IV (mt-CO2), F_0_F_1_-ATPase (mt-ATP6) and cytochrome B (mt-CYTB), increased with combination treatment only (Fig. 2E–G). Complex II (SDHB) transcription was not significantly altered by any treatment (Fig. 2H). Metformin and SB431542 appear to upregulate distinct sets of ETC genes, and when given jointly, the two treatments result in an additive increase in ETC gene transcription, suggesting that the treatments may control ETC gene transcription through distinct pathways.

**FIG. 2.**
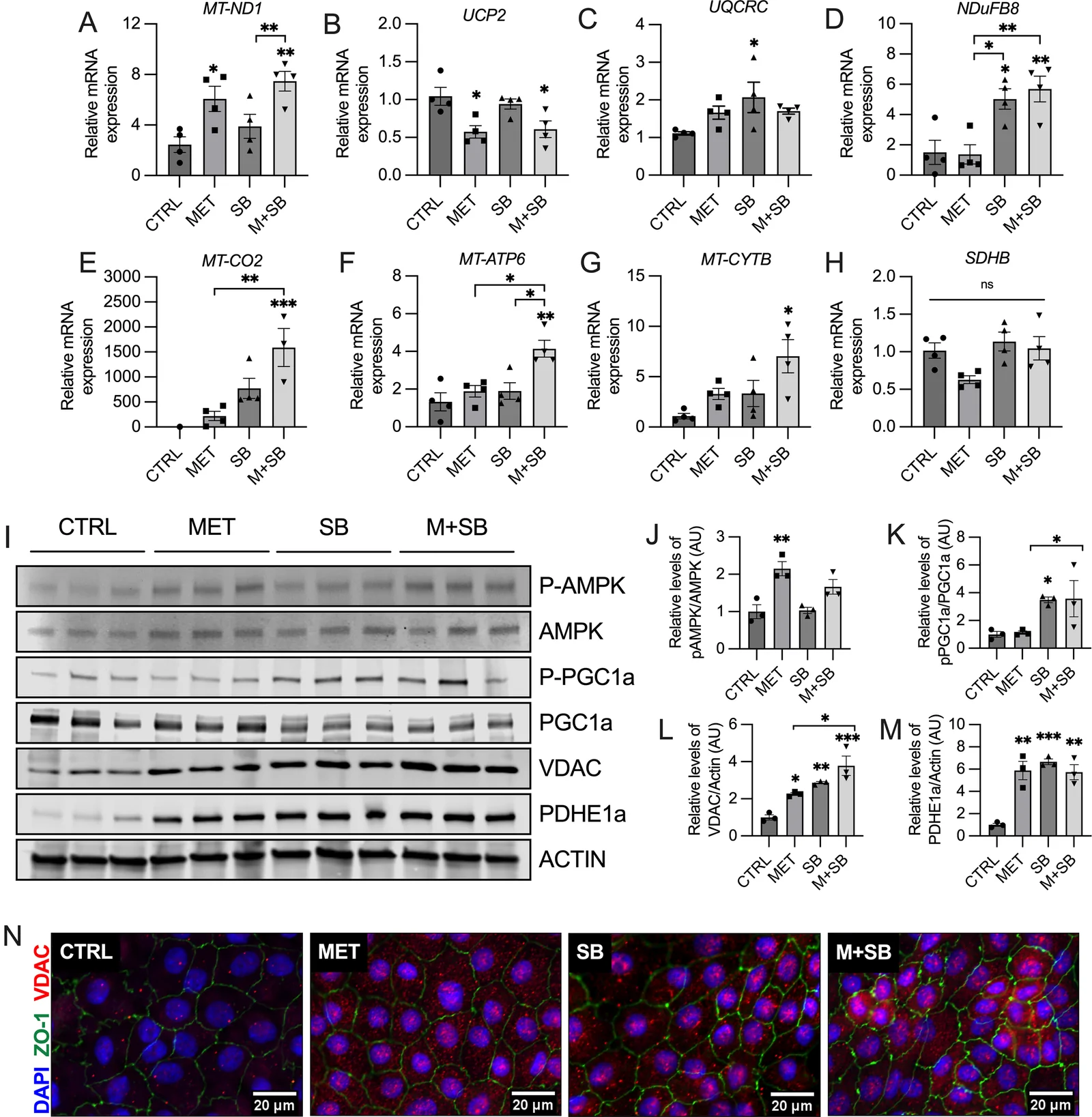
Metformin and SB431542 increase transcription of electron transport chain (ETC) genes. **(A)** RNA expression of mitochondrial-encoded complex I (MT-ND1), **(B)** uncoupling protein 2 (UCP2); **(C)** nuclear-encoded Complex III (UQCRC), **(D)** nuclear-encoded Complex I (NDuFB8), **(E)** mitochondrial-encoded Complex IV (MT-CO2), **(F)** mitochondrial-encoded F_0_-F_1_ ATPase (MT-ATP6), **(G)** mitochondrial-encoded Cytochrome B (MT-CYTB), and **(H)** nuclear-encoded Complex II (SDHB) (*n* = 4). **(I)** Western blot of phospho-AMPK, total AMPK, phospho-PGC1α, PGC1α, voltage-dependent anion channel (VDAC), PHDE1α, and β-actin; **(J)** Protein densitometry analysis of AMPK phosphorylation, **(K)** PGC1α phosphorylation, **(L)** Total VDAC, and **(M)** Total PDHE1α (*n* = 3). **(N)** Immunofluorescent staining of DAPI (*blue*), zonula occludens-1 (*green*), and VDAC (*red*). Data are presented as mean ± s.e.m. Asterisks above a sample denote statistical significance between that sample and the control group. *p < 0.05, **p < 0.01, ***p < 0.001. AMPK, AMP-activated protein kinase.

To further investigate whether the mechanism by which metformin and TGF-β inhibition increase directional solute transport in tubule cells is related to mitochondrial energy production, we measured expression and phosphorylation of enzymes and transcription factors linked to oxidative phosphorylation. Total AMPK expression increased additively with treatments, while combination treatment increased AMPK phosphorylation (Fig. 2I, J).

Pyruvate dehydrogenase E1-alpha (PDHE1α) catalyzes the conversion of pyruvate to acetyl-CoA and CO_2_ and provides the primary link between glycolysis and the TCA cycle. VDAC governs the entry and exit of mitochondrial metabolites.^17^ PDHE1α and VDAC protein expression increased significantly with all treatments (Fig. 2L, M). We examined mitochondrial architecture by staining control and treated cells for VDAC, which outlines mitochondrial shape (Fig. 3M). VDAC immunofluorescence labeling is sparse and punctate in control cells but increases in density and intensity with treatments (Fig. 2M). The increase in VDAC density and continuity is consistent with a reduction in mitochondrial fragmentation.

**FIG. 3.**
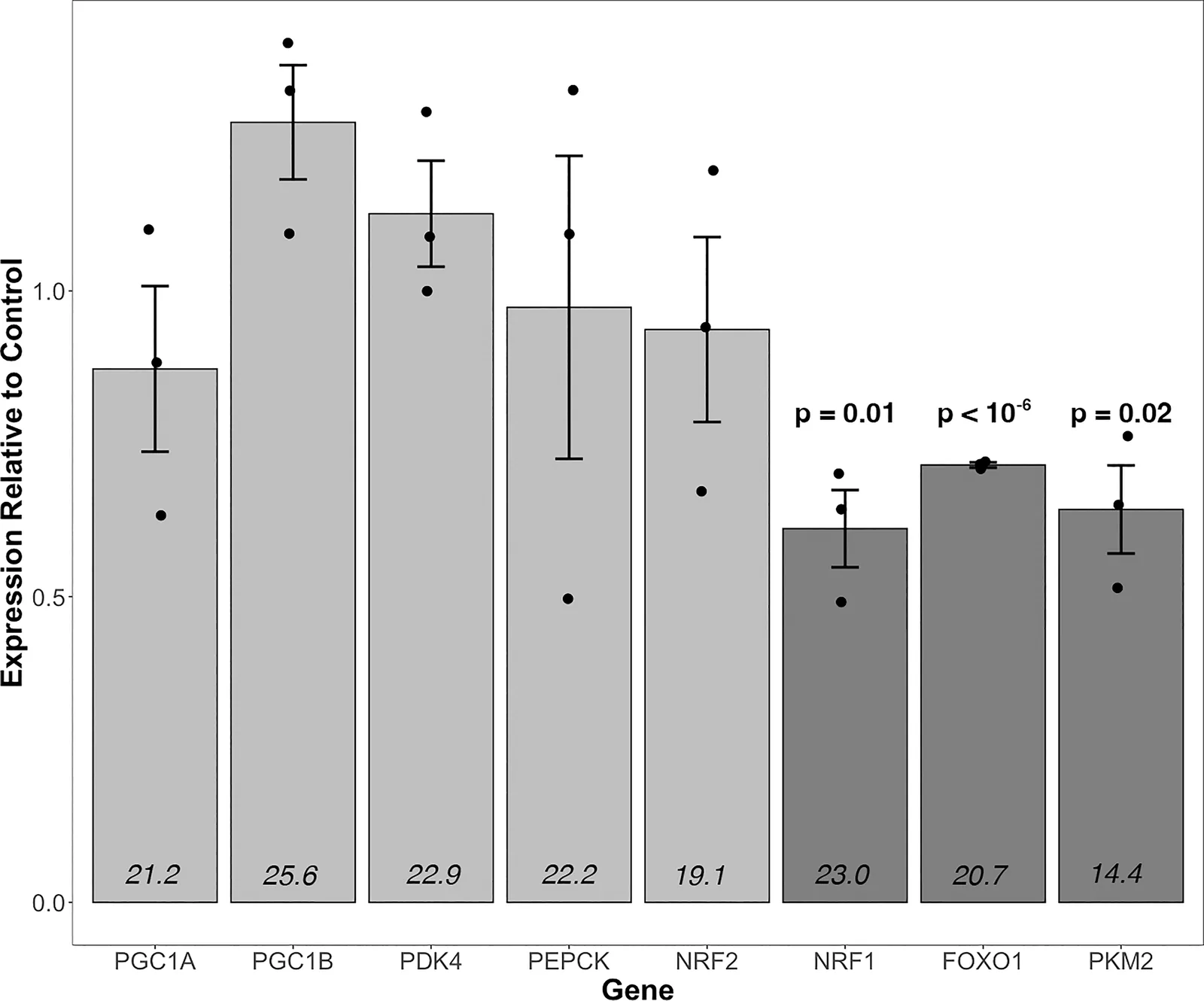
Transcription of PGC-1α, PGC-1β, and six PGC-1α-regulated genes between control and combination-treated renal proximal tubule cells. Messenger RNA levels of PGC-1α, PGC-1β, pyruvate dehydrogenase kinase 4 (PDK4), phosphoenolpyruvate carboxykinase (PEPCK), and nuclear regulatory factor 2 (NRF2) were not significantly different between control and combination-treated cells (light gray bars). In contrast, mRNA levels of nuclear regulatory factor 1 (NRF1), Forkhead box protein O1 (FOXO1), and pyruvate kinase M2 (PKM2) were significantly lower in combination-treated cells after Bonferroni correction for multiple comparisons (*dark gray bars*). Numbers in each bar denote polymerase chain reaction cycle number. *P*-values for statistically significant differences appear above each bar.

PGC-1α is regulated at transcription and posttranscriptional levels, including phosphorylation sites at threonine-177, serine-538, and serine 572.^18^ Activated AMPK phosphorylates peroxisome proliferator-activated receptor gamma coactivator-1α (PGC-1α) at threonine-177 and serine-538, which regulates the expression of proteins involved in fatty acid uptake and oxidation.^19^ Serine-572 is phosphorylated by Akt and inhibits PGC-1α cotranscriptional activity.^18^ PGC-1α phosphorylation at serine 572 increased significantly with SB431542 and combination treatments (Fig. 2K). In the absence of specific antibodies to phosphorylated PGC-1α at threonine-177 and serine-538, we surveyed transcription of PGC-1α, PGC-1β, and six genes that are transcriptionally regulated by PGC-1α to assess PGC-1α activity in control- and combination-treated cells. Messenger RNA levels of PGC-1α, PGC-1β, pyruvate dehydrogenase kinase 4 (PDK4), phosphoenolpyruvate carboxykinase (PEPCK), and nuclear regulatory factor 2 (NRF2) were not significantly different between control and combination-treated cells (Fig. 3). In contrast, mRNA levels of nuclear regulatory factor 1 (NRF1), Forkhead box protein O1 (FOXO1), and pyruvate kinase M2 (PKM2) were significantly lower in combination-treated cells after Bonferroni correction for multiple comparisons (Fig. 3).

### TGF-β inhibition increases expression of genes related to fatty acid oxidation

We assessed whether the treatments altered the transcription of genes associated with fatty acid metabolism. SB431542 increased transcription of Cluster of Differentiation 36 (CD36), the primary translocase that governs cellular fatty acid import (Fig. 4A). All treatments significantly increased the transcription of fatty acid binding protein 1 (FABP1), a carrier protein involved in fatty acid transport (Fig. 4B). SB431542 and combination treatment increased the expression of mitochondrial enzyme carnitine palmitoyl transferase 2 (CPT2), which governs mitochondrial fatty acid import (Fig. 4C). Protein expression of CD36 and FABP1 expression increased with SB431542 (Fig. 4D–F), while CPT2 protein expression significantly increased with combination treatment (Fig. 4G).

**FIG. 4.**
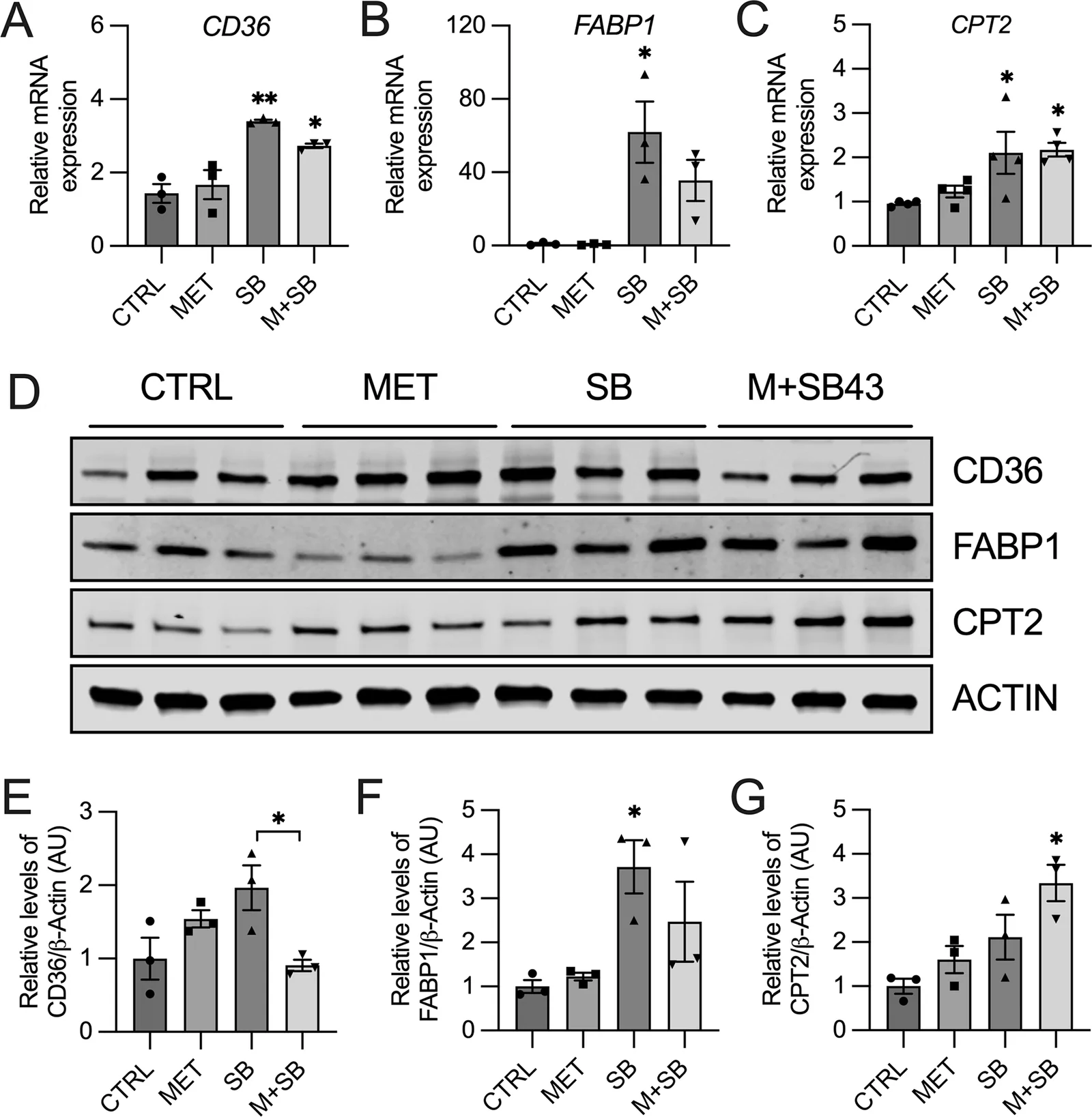
SB431542 induces expression of FAO genes. RNA expression of **(A)** cluster of differentiation 36 (CD36), **(B)** fatty acid binding protein 1 (FABP1), and **(C)** carnitine palmitoyl transferase 2 (CPT2). **(D)** Western blot protein analysis of CD36, FABP1, CPT2, and loading control β-actin. **(E)** Protein densitometry of data in panel D relative to loading control β-actin: **(F)** CD36 expression, **(G)** FABP1 expression, **(H)** CPT2. Data are presented as mean ± s.e.m. (*n* ≥ 3). Asterisks above a sample denote statistical significance between that sample and the control group. *p < 0.05, **p < 0.01, ***p < 0.001. FAO, fatty acid oxidation.

### Metformin and SB431542 additively increase mitochondrial respiratory capacity

To determine if changes in mitochondrial gene expression were accompanied by changes in mitochondrial function, we assessed the relative contribution of various ETC complexes to cellular respiration. The test operates by sequentially exposing cells to compounds that interfere with the function of ETC Complexes I, III, and V and uncoupling the mitochondrial proton gradient. Changes in cellular OCR can isolate contributions from cell proton leak and the basal-, maximal-, spare-, ATP-linked-, and nonmitochondrial respiration. We used the Complex I inhibitor rotenone, the Complex III inhibitor antimycin A, the Complex V inhibitor oligomycin, and the mitochondrial uncoupler CCCP (Fig. 5A, B). When normalized to cell number, basal respiration rate increased by 54.9% in SB431542-treated cells (control: 24.4 ± 1.3 pmol O_2_/min/10^4^ cells vs SB431542: 36.5 ± 1.8 pmol O_2_ × min^−1^/10^4^ cells [*p* < 0.0001]). Basal OCR was 53.1% lower in combination-treated cells (control: 24.4 ± 1.3 pmol O_2_ × min^−1^/10^4^ cells vs combination: 11.46 ± 1.6 pmol O_2_ × min^−1^/10^4^ cells [*p* < 0.0001], Fig. 5Ci), while metformin did not significantly affect basal OCR. CCCP may be used to uncouple mitochondrial respiration and determine cell maximal respiration capacity. Metformin and combination treatments significantly increased maximal respiration upon exposure to CCCP (Fig. 5B). Spare respiratory capacity, which measures a cell’s capacity to respond to rising energy demands or stress, is defined as the difference between maximal OCR and basal OCR. When compared with control cells, metformin and combination treatments improved spare respiratory capacity (control: 42.6 ± 3.9 pmol O_2_ × min^−1^/10^4^ cells vs metformin: 120.0 ± 5.5 pmol O_2_ × min^−1^/10^4^ cells [*p* < 0.0001], combination: 86.0 ± 3.6 pmol O_2_ × min^−1^/10^4^ cells [*p* < 0.0001] Fig. 5Cii).

**FIG. 5.**
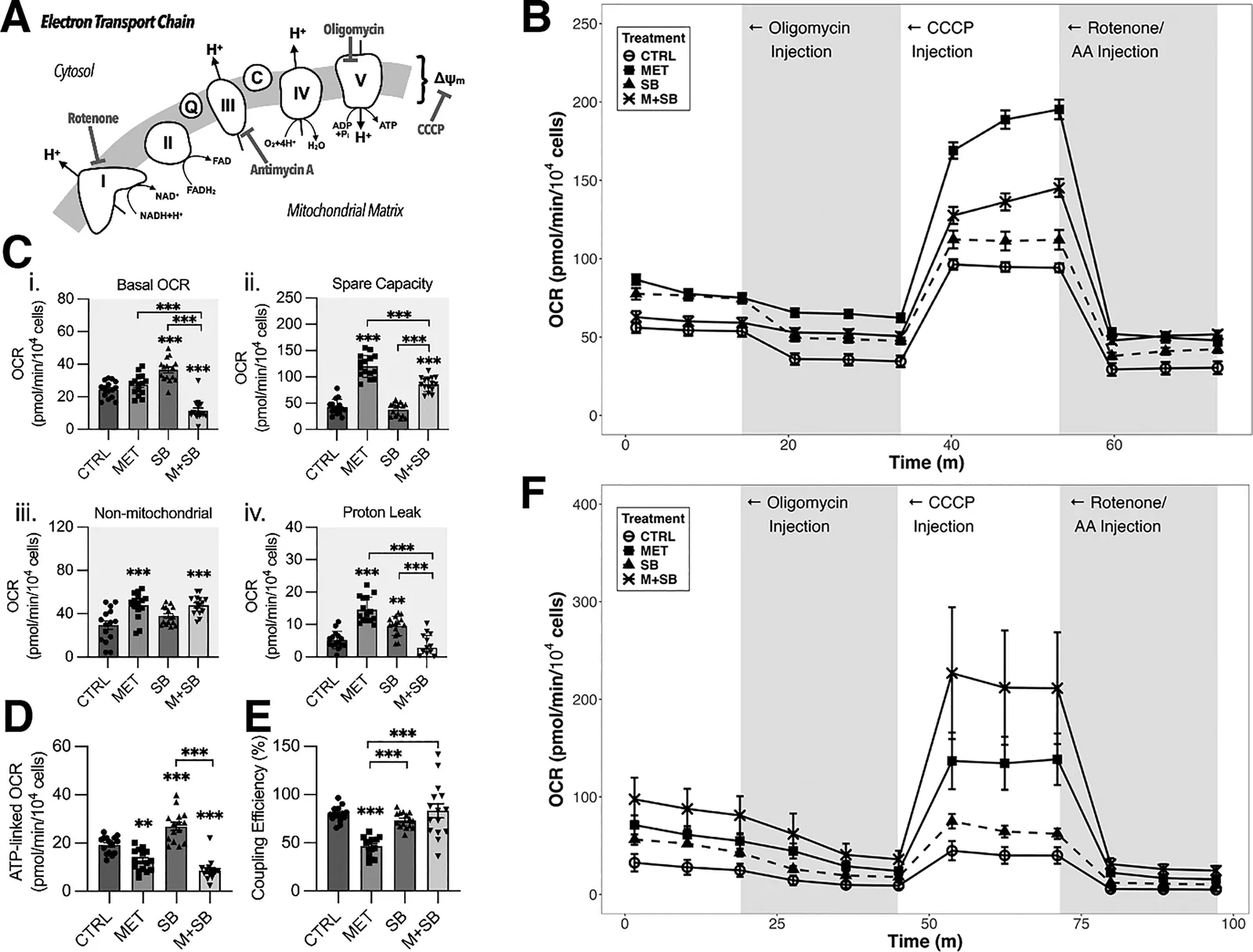
Synergistic effect on mitochondrial respiration of metformin and SB431542. **(A)** The electron transport chain (ETC) and the respiratory inhibitors used to assess cell respiratory capacity. **(B)** Mitochondrial stress test revealing divergent effects of metformin and SB431542 on oxygen consumption rates (OCR). **(C) i.** Basal OCR, indicating baseline oxidative phosphorylation. **ii.** Spare respiratory capacity, reflecting the cell’s ability to meet energetic demands (calculated as the difference between maximal OCR and basal OCR). **iii.** Nonmitochondrial OCR, measuring cytosolic oxygen consumption after ETC inhibition. **iv.** Proton leak, representing the proportion of basal respiration not linked to ATP production. **(D)** ATP-linked OCR, indicating the amount of basal respiration linked to ATP production. **(E)** Coupling efficiency (%), representing the proportion of basal OCR linked to ATP production. **(F)** Mitochondrial stress test after 46 weeks in culture, showing that TGF-β inhibition and metformin caused durable increases in oxygen consumption after very prolonged culture. Data are presented as mean ± s.e.m. (*n* = 15 at 2.5 weeks and *n* = 3 at 46 weeks). Asterisks above a sample denote statistical significance between that sample and the control group. *p < 0.05, **p < 0.01, ***p < 0.001. TGF-β, transforming growth factor-β.

Oligomycin, which stops ATP synthesis by inhibiting the F_0_ unit of F_0_F_1_-ATPase, was used to evaluate leak and ATP-linked respiration. The term “leak respiration” refers to the difference between respiration that occurs in the presence of oligomycin and nonmitochondrial respiration. When compared with control cells, proton leak increases about threefold with metformin and roughly twofold with SB431542 but is not significantly affected when the two treatments are combined (control: 5.2 ± 0.7 pmol O_2_ × min^−1^/10^4^ cells vs metformin: 15.0 ± 1.0 pmol O_2_ × min^−1^/10^4^ cells [*p* < 0.0001]; SB431542: 9.6 ± 1.0 pmol O_2_ × min^−1^/10^4^ cells [*p* = 0.0012]; combination: 2.88 ± 1.0 pmol O_2_ × min^−1^/10^4^ cells [*p* = 0.2982], Fig. 5Civ).

ATP-linked respiration may be calculated as the difference between the OCR before and after an injection of oligomycin. ATP-linked respiration decreased 33.6% with metformin and 55.5% with combination treatment but increased 39.6% with SB43152 (control: 19.25 ± 0.9 pmol O_2_ × min^−1^/10^4^ cells vs metformin: 12.79 ± 1.1 pmol O_2_ × min^−1^/10^4^ cells [*p* = 0.0008], SB431542: 26.88 ± 1.7 pmol O_2_ × min^−1^/10^4^ cells [*p* = 0.0036], combination: 8.574 ± 1.1 pmol O_2_ × min^−1^/10^4^ cells [*p* < 0.0001], Fig. 5D). Coupling efficiency is defined as the ratio between oxygen consumed to drive ATP synthesis to that used to cause proton leak, normalized to basal OCR. When compared with control cells, metformin reduced coupling efficiency (control 79.51 ± 2.05% vs metformin: 46.33 ± 2.58% [*p* < 0.0001]). SB431542 and combination treatment, however, did not significantly affect coupling efficiency compared with untreated cells (Fig. 5E). The increased oxygen consumption in combination-treated cells at baseline and at maximal respiration compared with control cells was present in cells continuously cultured for 46 weeks (Fig. 5F). We measured ECAR between conditions as a functional metric of glycolysis, and ECAR did not differ between control and SB431542-treated cells, while addition of metformin did subtly increase ECAR (data not shown). This effect of metformin has been reported in other cell types that typically rely on fatty acids.^20^ Whether tubule cells were able to use stored glycogen for glycolysis in this experiment and whether that contributed to differences in respiration is unknown.

### Metformin and SB431542 influence renal tubule epithelial cell substrate utilization

Substrate dependency describes the relative contributions of sugars, fats, and amino acids to cellular energy production. We assessed the effect of metformin and TGF-β inhibition on cell utilization of glucose, fatty acids, and glutamine using a series of respiratory inhibitors (Fig. 6A–C). Mitochondrial pyruvate carrier inhibitor UK5009 was used to inhibit glucose oxidation (Fig. 6A). Glutaminase 1 (GLS1) inhibitor BPTES was used to inhibit glutamine oxidation (Fig. 6B). Carnitine palmitoyl transferase 1 (CPT1) inhibitor etomoxir was used to inhibit fatty acid oxidation (Fig. 6C). After inhibition of the target pathway, the two remaining inhibitors were added to suppress the remaining oxidation pathways. Target pathway dependency was then calculated as a percentage of the total inhibition achieved by all three inhibitors (Fig. 6D, F). Metformin and combination treatment significantly reduced OCR glucose dependency (Fig. 6D). No treatment significantly changed OCR glutamine dependency, although metformin increased glutamine OCR dependency in the context of SB431542 (Fig. 6E).

**FIG. 6.**
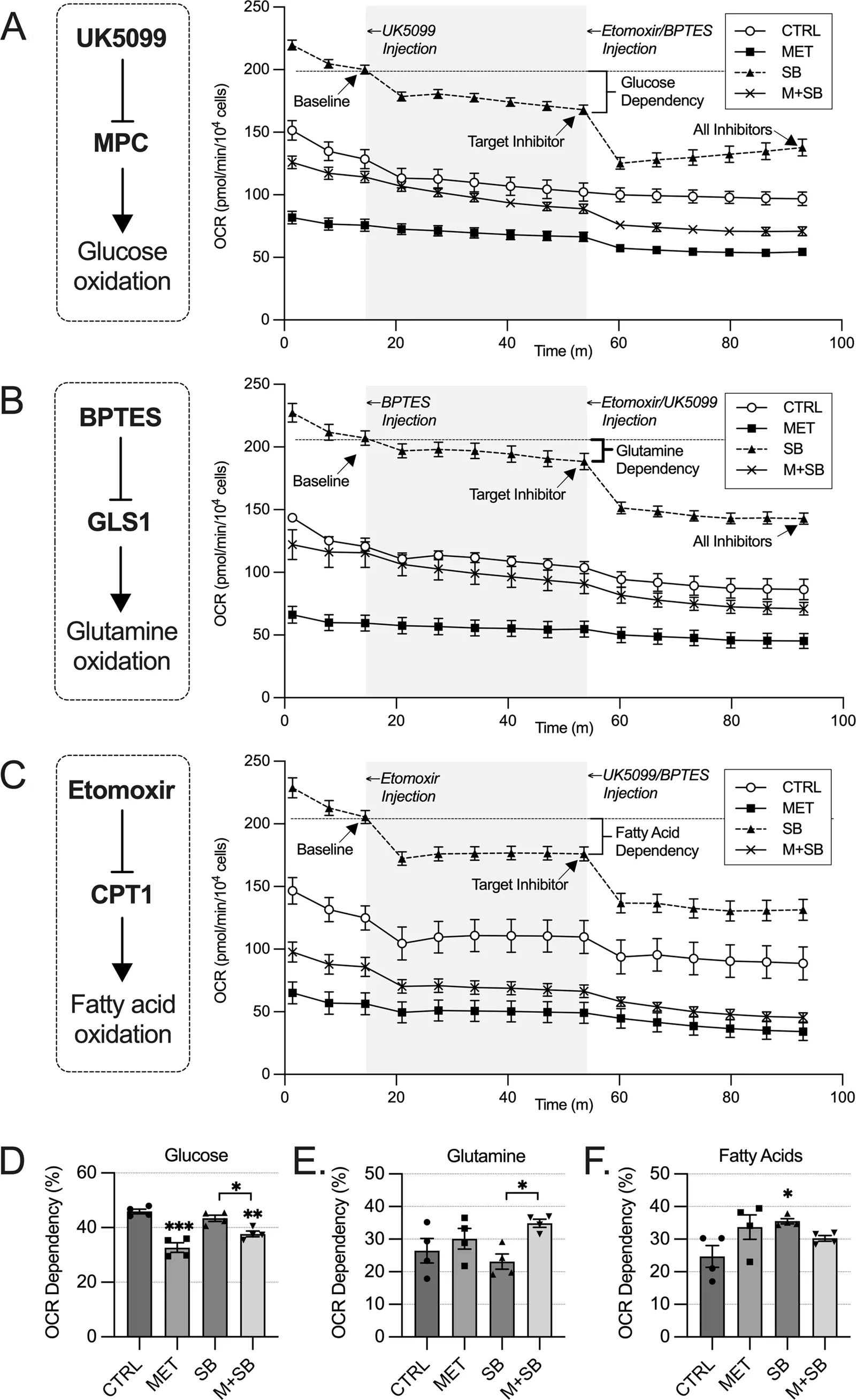
Metformin reduces glucose dependency, while SB431542 increases fatty acid oxidation (FAO). **(A)** Glucose dependency test. **(B)** Glutamine dependency test. **(C)** Fatty acid dependency test. **(D)** Glucose oxygen consumption rate (OCR) dependency. **(E)** Glutamine OCR dependency. **(F)** Fatty acid OCR dependency. Data are presented as mean ± s.e.m. (*n* = 4). Asterisks above a sample denote statistical significance between that sample and the control group. *p < 0.05, **p < 0.01, ***p < 0.001.

## Discussion

The dedifferentiation due to cell culture stress is a major barrier to medical use of cells *in vitro* and a major focus of our present work developing a biohybrid artificial kidney. We reconfirmed that inhibition of TGF-βR1 with SB431542 increased apicobasal transport by cultured primary cells and that this effect persists for up to 46 weeks in continuous culture. Apicobasal transport is ultimately driven by basolateral Na-K-ATPase and consumes one ATP molecule for each three sodium ions exchanged for two potassium ions. We examined whether the genes involved in oxidative phosphorylation also changed in response to metformin and TGF-β. Complex I of the ETC has multiple subunits encoded by both nuclear and mitochondrial DNA. We found evidence that both mitochondrial and nuclear transcription is upregulated by metformin and by SB431542, respectively.

Complex II did not seem affected by either treatment, but transcripts for other mitochondrially encoded proteins in the ETC were additively increased by metformin and by SB431542. We tentatively interpret the increased transcript count of mitochondrially encoded genes, consistent with an increase in mitochondrial number as previously reported.^3^ Indirect immunofluorescence imaging of the VDAC shows increased signal in combination-treated cells, again consistent with increased number and/or volume of mitochondria. Mitochondrial biogenesis is a coordinated response to energy demand closely linked to a transcription factor, peroxisome proliferator-activated receptor gamma coactivator 1-alpha (PGC-1α). Notably, Smad3 can downregulate PGC-1α expression by directly binding to its enhancer influence. Furthermore, PGC-1α is activated by AMPK, suggesting a unifying mechanism for the additive effects of metformin and TGF-β blockade. Thus, we measured PGC1α expression to test whether SB431542 might influence Smad3-induced PGC-1α repression.^21^ Finally, both metformin and SB431542 increased PDHE1α protein levels. PDH catalyzes the conversion of pyruvate to acetyl-CoA and connects cytosolic glycolysis to the TCA in mitochondria. Expression of PDHE1α is regulated again by PGC1α, consistent with a programmatic response of the cell to increased energy demand despite apparently little change in PGC1α concentrations in our experiments. There was no difference in PGC1α mRNA or protein level between control and treated cells, in contrast to results from other studies, which show that AMPK increases PGC1α transcription.^22^ Furthermore, transcriptional profiles of PGC-1α-regulated genes were unchanged or lower in treated cells, arguing against our hypothesis that PGC-1α was responsible for the increased mitochondrial respiration. However, interpretation of PGC1α expression data here should be tempered by the recognition that hyperinsulinemia represses PGC1α expression and there is significant insulin supplementation in cell culture media.^23^

Tubule cells *in vivo* preferentially use fatty acids as a source of energy and typically contribute both reabsorbed and newly synthesized glucose to the circulation. In contrast, cell culture media typically contains excess glucose and insulin, the latter possibly as a low-cost surrogate for insulin-like growth factor-1. We probed whether the increased apicobasal transport we observed induced any changes in the *in vitro* substrate utilization by primary renal tubule cells. Indeed, we observed increased transcript and protein concentrations of fatty acid translocase (CD36), fatty acid binding protein (FABP), and carnitine palmitoyltransferase II (CPT2). Thus, all steps involved in fatty acid metabolism—including cellular uptake (CD36), cytosolic stabilization (FABP), and mitochondrial import (CPT2)—were upregulated by TGF-β inhibition.

Renal tubule cells grown in conventional culture differ from cells in a healthy kidney in morphology, function, and metabolism. The *in vitro* switch from oxidative phosphorylation to aerobic glycolysis bears some resemblance to both the Warburg and Crabtree effects.^24^ These two related effects in which cells depart from healthy metabolism are accompanied by widespread changes in morphology, function, and gene expression profile similar to patterns we observe when culturing proximal tubule cells *in vitro*. The Crabtree effect may be more evident in renal proximal tubule cells than in other kidney cell types, as these cells are more dependent on oxidative phosphorylation and indeed produce rather than consume glucose.^25,26^ This perspective may have explanatory power for some of the profound differences we observe between *in vivo* (locally hypoglycemic) and *in vitro* (hyperglycemic) phenotypes.

Our team has worked to miniaturize Humes’ Renal Assist Device into an implanted bioartificial kidney with synthetic filters and living cells.^27^ Most recently, we have focused on identifying and mitigating the factors that cue tubule cells to dedifferentiate under the stress of artificial culture.^3,4^ Here, we redemonstrate that specific inhibition of TGF-β signaling restores primary (i.e., untransformed) tubule cell apicobasal transport and extend that observation out to 46 weeks in continuous culture. Furthermore, combined treatment with metformin and a TGF-β inhibitor increases oxidative phosphorylation and appears to increase utilization of fatty acids rather than glucose. These data support the notion that these interventions can increase transporter expression *in vitro* and may help mitigate some aspects of cell culture stress. These findings suggest that TGF-β signaling inhibition and metformin treatment may improve aspects of mitochondrial function and transporter expression in cultured tubule cells, potentially increasing their relevance for research and renal cell therapy.

## Authors’ Contributions

K.H. and W.H.F. conceived and designed research. K.H. and A.I. performed experiments, analyzed data, interpreted results of experiments, prepared figures, and drafted article. K.H., S.R., R.Z., and W.H.F. edited, revised, and approved the final version of article.

## Supplemental Material

sj-pdf-1-tea-10.1177_19373341251389007 — Supplemental material for Inhibition of TGF-β in Tubule Cells Increases Respiration via Upregulation of Respiratory Genes Supplemental material, sj-pdf-1-tea-10.1177_19373341251389007 for Inhibition of TGF-β in Tubule Cells Increases Respiration via Upregulation of Respiratory Genes by

## Data Availability

All relevant data can be found within the article and its supplementary information.
