# Supplementary material for: Inhibition of TGF-β in Tubule Cells Increases Respiration via Upregulation of Respiratory Genes
Source: Tissue Eng Part A. 2025 Oct 22;32(15-16):559–69. doi: 10.1177/19373341251389007 (PMC13656296; doi:10.1177/19373341251389007)
Supplement: Supplementary file 1 [file sj-pdf-1-tea-10.1177_19373341251389007.pdf]

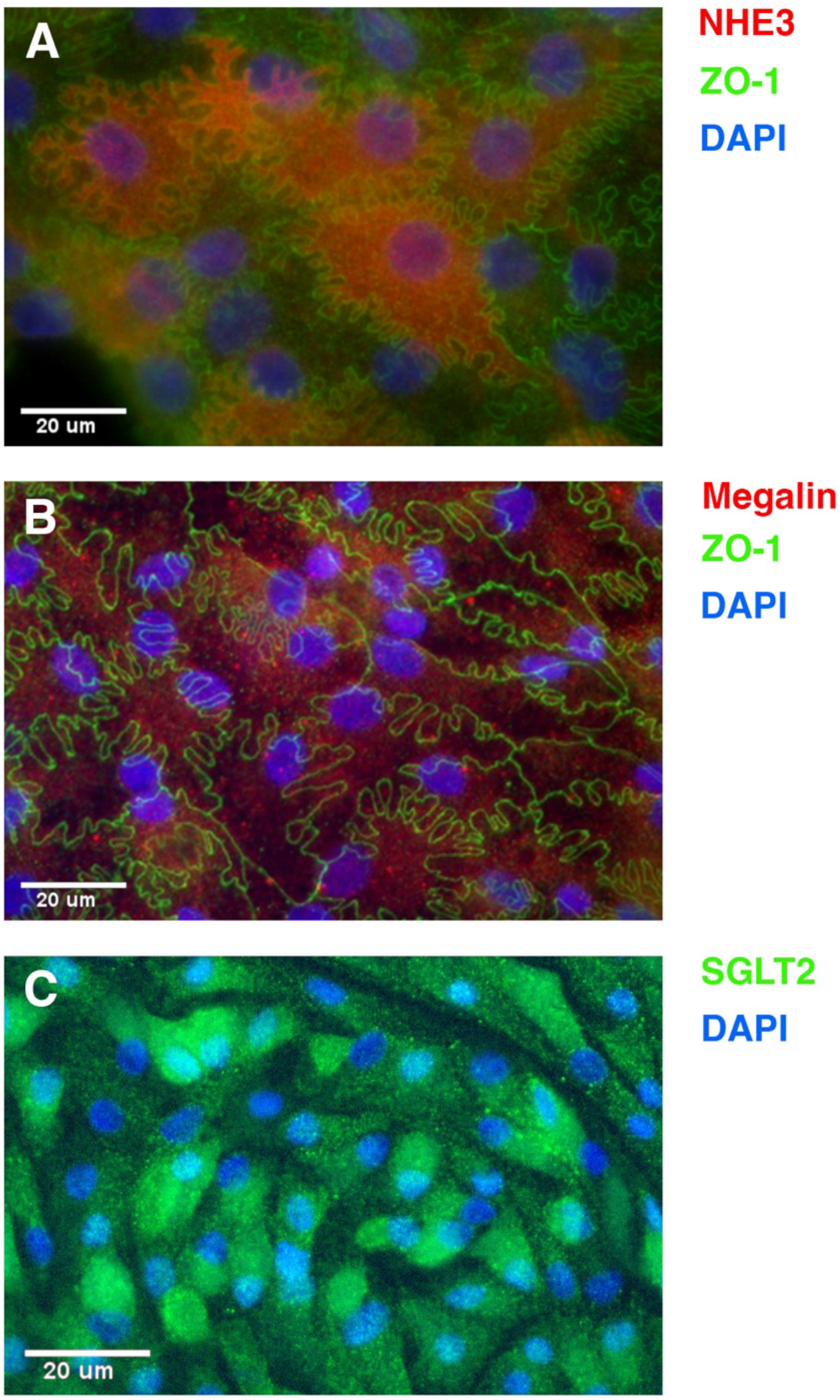

Supplemental Figure 1: Cultured primary RPTECs express membrane proteins characteristic of renal proximal tubule cells. A, cells express NHE3 (red). B, cells express megalin (red). C. Cells express SGLT2 (green).

Table S1: RT-PCR primers.

Target	Forward (5' to 3')	Reverse (5' to 3')
CD36	GGCTGTGACCGGAACTGTG	AGGTCTCCAACTGGCATTAGAA
CPT2	CATACAAGCTACATTTCGGGACC	AGCCCGGAGTGTCTTCAGAA
FABP-1	GTGTCGGAAATCGTGCAGAAT	GACTTTCTCCCCTGTCATTGTC
FOXO1	GGATGTGCATTCTATGGTGTACC	TTTCGGGATTGCTTATCTCAGAC
MT-ATP6	TAGCCATACACAACACTAAAGGACGA	GGGCATTTTTAATCTTAGAGCGAAA
MT-CO2	CTGAACCTACGAGTACACCG	TTAATTCTAGGACGATGGGC
MT-CYTB	ATCACTCGAGACGTAAATTATGGCT	TGAACTAGGTCTGTCCCAATGTATG
MT-ND1	CCACCTCTAGCCTAGCCGTTTA	GGGTCATGATGGCAGGAGTAAT
NDuFB8	ACAGGAACCGTGTGGATACAT	CCCCACCCAGCACATGAAT
NRF1	GCTGATGAAGACTCGCCTTCT	TACATGAGGCCGTTTCCGTTT
NRF2	TCAGCGACGGAAAGAGTATGA	CCACTGGTTTCTGACTGGATGT
PDK4	GGAGCATTTCTCGCGCTACA	ACAGGCAATTCTTGTCGCAAA
PEPCK	GCCATCATGCCGTAGCATC	AGCCTCAGTTCCATCACAGAT
PGC-1a	TCTGAGTCTGTATGGAGTGACAT	CCAAGTCGTTCACATCTAGTTCA
PGC-1b	GATGCCAGCGACTTTGACTC	ACCCACGTCATCTTCAGGGA
PKM2	ATGTCGAAGCCCCATAGTGAA	TGGGTGGTGAATCAATGTCCA
SDHB	ACAGCTCCCCGTATCAAGAAA	GCATGATCTTCGGAAGGTCAA
UCP2	GGAGGTGGTCGGAGATACCAA	ACAATGGCATTACGAGCAACAT
UQCRC	TTCAGCAATTTAGGAACCACCC	GGTCACACTTAATTTGCCACCAA

Table S2: Primary antibodies.

Target	Manufacturer	Catalog number
Actin	Santa Cruz	sc-47778
AMPK	Invitrogen	MA5-15815
CD36	ProteinTech	18836-1-AP
CPT2	ProteinTech	26555-1-AP
FABP-1	Invitrogen	PA5145248
PDHE-1a	Abcam	ab110330
PGC-1a	Abcam	ab72230
Phospho-AMPK	Cell Signaling	2535
Phospho-PGC-1a	R&D Systems	AF6650
VDAC	Cell Signaling	4661
ZO-1	Invitrogen	33-9100
